# Baseline and usual cardiorespiratory fitness and the risk of chronic kidney disease: A prospective study and meta-analysis of published observational cohort studies

**DOI:** 10.1007/s11357-023-00727-3

**Published:** 2023-01-17

**Authors:** Setor K. Kunutsor, Nzechukwu M. Isiozor, Jonathan Myers, Samuel Seidu, Kamlesh Khunti, Jari A. Laukkanen

**Affiliations:** 1 Diabetes Research Centre, University of Leicester, Leicester General Hospital, Gwendolen Road, Leicester, LE5 4WP UK; 2 National Institute for Health Research Bristol Biomedical Research Centre, University Hospitals Bristol NHS Foundation Trust and University of Bristol, Bristol, UK; 3 Musculoskeletal Research Unit, Translational Health Sciences, Bristol Medical School, University of Bristol, Learning & Research Building (Level 1), Southmead Hospital, Bristol, BS10 5NB UK; 4 Institute of Clinical Medicine, Department of Medicine, University of Eastern Finland, Kuopio, Finland; 5 Cardiology Division, Veterans Affairs Palo Alto Healthcare System and Stanford University, Palo Alto, CA USA; 6 Central Finland Health Care District Hospital District, Department of Medicine,Jyväskylä, Finland District, Jyväskylä, Finland; 7 Institute of Public Health and Clinical Nutrition, University of Eastern Finland, Kuopio, Finland

**Keywords:** Cardiorespiratory fitness, physical activity, chronic kidney disease, cohort study, meta-analysis

## Abstract

Chronic kidney disease (CKD) is a global public health burden but is largely preventable. Few studies have reported an association between high levels of cardiorespiratory fitness (CRF) and decreased risk of CKD. However, some relevant aspects of the association, such as the dose-response relationship and the long-term relevance of CRF levels to CKD, have yet to be addressed. We, therefore, aimed to quantify the nature and magnitude of the association between CRF and CKD in a cohort of 2099 men aged 42-61 years with normal kidney function at baseline. CRF was directly measured using a respiratory gas exchange analyzer during cardiopulmonary exercise testing. Hazard ratios (HRs) (95% CIs) were estimated for the incidence of CKD. Correction for within-person variability was made using data from repeat measurements of CRF taken 11 years after baseline. A total of 197 CKD events were recorded during a median follow-up of 25.8 years. The age-adjusted regression dilution ratio for CRF was 0.59 (95% CI: 0.53-0.65). The risk of CKD decreased in a graded fashion with increasing CRF. Comparing extreme tertiles of CRF, the HR (95% CI) for CKD was 0.67 (0.46-0.97) following adjustment for several established and emerging risk factors. The corresponding adjusted HR (95% CI) was 0.51 (0.27-0.96) following correction for within-person variability. In a meta-analysis of five population-based cohort studies (including the current study) comprising 32,447 participants and 4,043 CKD cases, the fully-adjusted risk ratios (95% CIs) for CKD comparing extreme tertiles of baseline and long-term CRF values were 0.58 (0.46-0.73) and 0.40 (0.27-0.59), respectively. Findings from a new prospective study and pooled analysis of previous studies plus the new study indicate that high CRF levels are strongly and independently associated with a reduced risk of CKD and consistent with a linear dose-response relationship. Using single baseline measurements of CRF to investigate the association between CRF and CKD risk could considerably underestimate the true association.

**Supplementary Information:**

The online version contains supplementary material available at 10.1007/s11357-023-00727-3.

## Introduction

Chronic kidney disease (CKD) is a significant cause of global morbidity and mortality and is associated with substantial costs to global economies and healthcare systems [1, 2]. The presence of CKD is associated with an increased risk of cardiovascular disease (CVD); it is also a risk multiplier in hypertension and diabetes [2, 3] which are major risk factors for CKD [4, 5]. In 2017, 1.2 million people died from CKD worldwide, making it the 12^th^ leading cause of death globally [6]. End-stage renal disease (ESRD) is a potential outcome of CKD and may require costly renal replacement therapy [2]. In the United States in 2019, treating Medicare beneficiaries with CKD cost $87.2 billion, and treating ESRD cost an additional $37.3 billion [7]. Due to population ageing and an increasing burden due to major risk factors such as hypertension and diabetes [4, 5], the prevalence and incidence of CKD continue to increase [2]. In 2017, the global prevalence of CKD was 9.1%, representing an increase of 29.3% since 1990 [6]. Though effective treatments exist for CKD, it is also largely preventable. Chronic kidney disease is a global public health burden, which warrants considerable attention in global health policy decision-making. There is a need to identify modifiable risk factors for CKD that have predictive relevance and could aid further in the development of effective preventive strategies, especially in those at high risk of developing CKD.

Physical activity and exercise training (a subset of physical activity that is structured with the

intent of developing physical fitness [8]) are well established to have many beneficial effects. These include a reduction in the risk of non-communicable diseases such as CVD, diabetes and hypertension as well as mortality [9–12]. Given that uncontrolled diabetes and hypertension increase the risk for CKD [4, 5], the beneficial effects of physical activity may also extend to preventing the incidence of CKD and slowing its progression [13]. Cardiorespiratory fitness (CRF) is an indicator of cardiopulmonary function and is often expressed as maximal oxygen uptake (VO_2max_) or peak VO_2_ (VO_2peak_); the gold standard of VO_2_ measurement is cardiopulmonary exercise testing (CPX) [14]. Though CRF is determined by many factors such as age, sex, health status and genetics, physical activity and exercise training remain the most established methods of increasing levels of CRF [15, 16]. Due to its strong inverse and independent association with adverse cardiovascular outcomes [17, 18], which is stronger than that of traditional risk factors such as diabetes and smoking [14, 19], CRF has recently been proposed as a vital sign [14]. Though a number of previous prospective studies have reported strong associations between increased CRF and decreased risk of CKD (with risk reductions ranging from 27 to 59%) [20–23], certain relevant aspects of the association such as the dose-response relationship and accounting for within-person variability in levels of CRF, were not addressed. It will be useful to know the range of CRF values for which the risk of CKD decreases and if a threshold exists. Furthermore, data on the extent to which CRF varies within individuals enhances the interpretation of epidemiological studies in an aetiological context. We have shown in previous studies that CRF exhibits high within-person variability [24–26], which could be the result of measurement errors, lifestyle changes, ageing, and development of chronic disease during long-term follow-up. Hence, analysis which only employs baseline measurements of CRF could underestimate the true strength of any aetiological association between CRF and disease outcome (i.e. “regression dilution bias” [27]). It is possible that previous estimates of the association between CRF and CKD risk may have been biased due to inability to correct for regression dilution bias [20–23].

To address the limitations of previous studies on the topic, we aimed to re-evaluate the nature and magnitude of the prospective association between CRF and CKD risk using a population-based prospective cohort of 2,099 men with no previous history of CKD from eastern Finland. We conducted dose-response analysis and repeat measurements of CRF performed several years apart in a random sample of participants enabled correction for regression dilution bias. Finally, we conducted a pooled analysis of the previous studies that have evaluated the prospective association between baseline CRF levels and CKD risk. This enabled us to (i) overcome sample size limitations of individual studies; (ii) increase precision; (iii) minimise any bias; and (iv) draw more reliable conclusions about the association between CRF and CKD risk.

## Materials and methods

### Study design and participants

We conducted the primary cohort study in accordance with STROBE (STrengthening the Reporting of OBservational studies in Epidemiology) guidelines for reporting observational studies in epidemiology (Electronic Supplementary Material 1). The participants included in the current analysis were recruited into the Kuopio Ischemic Heart Disease (KIHD) study, a general population-based prospective cohort study designed to evaluate the role of established and emerging risk factors for CVD and other chronic disease outcomes among the Finnish population. Details of the study design and recruitment methods have been described in previous reports [28, 29]. Briefly, a representative sample of 3,433 men aged 42-61 years who were inhabitants of Kuopio city and its surrounding rural communities in eastern Finland were invited for screening which was carried out between March 1984 and December 1989. Of the 3,433 men, 3,235 were found to be potentially eligible and of this number, 2,682 provided consent to participate in the study and 553 did not respond to the invitation or declined to participate. For this analysis, we excluded men with (i) existing kidney disease at baseline (*n*=56) and (ii) missing data on the exposure or potential confounders (*n*=527) (Electronic Supplementary Material 2). This left a total of 2,099 men who had complete information on CRF, relevant covariates, and CKD events for the analyses (Electronic Supplementary Material 3). The Research Ethics Committee of the University of Kuopio approved all study procedures, which were conducted according to the Declaration of Helsinki. Each study participant provided written informed consent.

### Assessment of CRF

Cardiorespiratory fitness was assessed using VO_2peak_, which was directly assessed using a computerized metabolic measurement system (Medical Graphics, USA) during a maximal symptom-limited exercise-tolerance test on an electrically braked cycle ergometer conducted between 8:00 am and 10:00 am [30]. The standardized testing protocol included a 3-min warm-up at 50 watts (W; 1W = 6.12 kgm/min). This was followed by 20 W/min increases in workload with direct analyses of expired respiratory gases. Respiratory gas exchange was measured by the breath-by-breath method, which involved breath sample collection via a face-mask. The respiratory gas analyzer expressed VO_2peak_ as an average value recorded over 8 seconds. Peak oxygen uptake was defined as the highest or peak attained value for oxygen consumption, expressed as mL/kg/min; VO_2peak_ was also expressed in metabolic equivalents (METs) (1 MET is defined as the amount of oxygen consumed while sitting at rest and corresponds to an oxygen uptake of 3.5 mL/kg/min). The respiratory exchange ratio (RER), defined as the ratio between respiratory gases (V̇CO_2_ and VO_2_,) was obtained exclusively from ventilatory expired gas analysis. Maximal effort was defined as RER greater than or equal to 1.1 [31]. Repeat measurements of CRF were performed 11 years after baseline in a random subset of the study participants [25, 32, 33].

### Assessment of covariates

Physical measurements, blood biomarkers measurements, and assessment of lifestyle characteristics and medical history have been described in detail in previous reports [34]. Blood pressure was recorded by an experienced nurse with a random-zero sphygmomanometer (Hawskley, UK) between 8:00 and 10:00 AM. After a supine rest of 5-minutes, blood pressure was measured three times in a supine position, once in a standing position, and twice in a sitting position with 5-minute intervals, and the arithmetic mean of all available measurements was taken [35, 36]. Body mass index (BMI) was estimated as weight in kilograms divided by the square of height in meters. For measurements of blood biomarkers, participants were required to fast overnight and abstain from drinking alcohol for at least 3 days and from smoking for at least 12 hours before blood samples were taken between 8:00 am and 10:00 am. Serum samples were stored frozen at -80 °C before measurements of lipids and biochemical analytes. Fasting plasma glucose (FPG) was determined using fresh samples, which was measured using the glucose dehydrogenase method (Merck, Darmstadt, Germany) following protein precipitation by trichloroacetic acid. Self-administered lifestyle and health questionnaires were used to assess prevalent medical conditions, use of medications and lifestyle characteristics such as smoking, alcohol consumption, physical activity and socioeconomic status (SES) [37]. A history of coronary heart disease (CHD) was defined as previous myocardial infarction, angina pectoris, the use of nitroglycerin for chest pain ≥ once a week or chest pain. The assessment of SES involved the creation of a summary index comprising relevant indicators such as income, education, occupational prestige, material standard of living and housing conditions [38–40]. The composite SES index ranged from 0 to 25, with higher values indicating lower SES. Energy expenditure of physical activity was assessed using the validated KIHD 12-month leisure-time physical activity questionnaire [41, 42], modified from the Minnesota Leisure-Time physical activity Questionnaire [43].

### Ascertainment of incident CKD

Estimated glomerular filtration rate (GFR) was estimated using the Chronic Kidney Disease Epidemiology Collaboration (CKD-EPI) equation [44] using the formula: 141 x (creatinine in mg/dl / 0.9)^-1.209^ x 0.993^Age^. Chronic kidney disease was defined as kidney damage (e.g., albuminuria) or estimated GFR lower than 60 mL/min per 1.73 m^2^ (or both) for 3 months or longer based on the National Kidney Foundation Kidney Disease Outcomes Quality Initiative (KDOQI) guidelines [45]. In the KIHD study, participants are under continuous surveillance for the development of new outcomes including CKD cases. All incident CKD cases that occurred from study entry to 2014 were included. Chronic kidney disease outcomes were collected from the National Hospital Discharge Register data by computer linkage and a comprehensive review of available hospital records, wards of health centres, health practitioner questionnaires, and medico-legal reports. No losses to follow-up were recorded as all participants in the KIHD study (using Finnish personal identification codes) are under continuous surveillance for the development of new outcomes including CKD cases.

### Data analyses

#### Prospective cohort analyses

Skewed variables (alcohol consumption and physical activity) were log transformed to achieve approximately normal distributions. Baseline characteristics were presented as means (standard deviation, SD) or median (interquartile range, IQR) for continuous variables based on distribution of the data and counts (percentages) for categorical variables. Time-to-event analyses were conducted using Cox proportional hazard regression models after confirmation of no major departure from the proportionality of hazards assumptions using scaled Schoenfeld residuals [46]. To quantify and correct for within-person variability in CRF levels, which is the extent to which an individual’s CRF measurements vary around the long-term average exposure levels (“usual levels”) [47], an age-adjusted regression dilution ratio (RDR) was estimated by regressing available repeat measurements on baseline values [48]. The RDR assumes that the “usual levels” of CRF represent the true long-term exposure of CRF levels on CKD risk. To correct for regression dilution bias, the estimated disease association (log hazard ratio and its 95% confidence intervals) was divided by the RDR.

To explore a potential nonlinear dose-response relationship between CRF and CKD risk, we constructed a multivariable restricted cubic spline (RCS) with knots at the 5th, 35th, 65th, and 95th percentiles of the distribution of CRF as recommended by Harrell [49]. Cardiorespiratory fitness was modeled continuously (per 1 MET increase in CRF) and as categories (tertiles) defined according to the baseline distribution of CRF levels. Adjustment for covariates was based on two models: (Model 1) age and (Model 2) Model 1 plus systolic blood pressure (SBP), history of type 2 diabetes (T2D), smoking status, history of hypertension, history of CHD, total cholesterol, alcohol consumption, estimated GFR, physical activity, and SES. These covariates were selected based on the following: (i) their established roles as risk factors for CKD [4, 5], (ii) published associations with CKD in the KIHD study [50], or (iii) their potential as confounders based on known associations with CKD outcomes and observed associations with the exposure using the available data [51]. Formal tests of interaction were used to assess statistical evidence of effect modification by categories of pre-specified clinically relevant individual level characteristics.

### Systematic review and meta-analysis

We conducted a meta-analysis of published observational cohort studies that had reported on the association between CRF levels and risk of CKD, using a predefined protocol registered in the PROSPERO register (CRD42022333142). The review was conducted and reported in accordance with PRISMA and MOOSE guidelines [52, 53] (Electronic Supplementary Materials 4 and 5). We searched MEDLINE, Embase, and the “Cited Reference Search” function in Web of Science up to 22 May 2022 for published observational population-based cohort studies with at least one year follow-up that had evaluated the associations of CRF with risk of CKD. The computer-based searches combined MeSH search terms and free texts related to the exposure (e.g., “cardiorespiratory fitness”, “aerobic capacity”) and outcome (e.g., “chronic kidney disease”, “renal insufficiency”). No restrictions were placed on language or the publication date. The detailed search strategy is presented in Electronic Supplementary Material 6.

Two authors (SKK and N.M.I.) initially screened the titles and abstracts of the retrieved citations to assess their potential for inclusion. This was conducted using Rayyan (http://rayyan.qcri.org), an online bibliographic tool that helps to expedite the screening process using a process of semi-automation [54]. Two authors (S.K.K. and N.M.I.) independently performed full-text evaluation, data extraction, and risk of bias assessments. Discrepancies were discussed and consensus reached with involvement of a third author (J.A.L.). Information was extracted on study characteristics such as study design, publication year, geographical location, baseline age, duration of follow-up, sample size, CRF assessment method, number of CKD events, risk ratios for the most adjusted models, and covariates adjusted for. The risk of bias within individual observational studies was assessed using the Cochrane Risk of Bias in Non-randomised Studies – of Interventions (ROBINS-I) tool [55].

The summary measure of association was the relative risk (RR) with 95% CIs. To enable a consistent approach to the meta-analysis and enhance comparison with the primary analysis, reported study-specific risk estimates were also transformed to extreme tertiles of CRF using standard statistical methods [56, 57], which have been described in detail previously [58, 59]. The associations of “usual levels” of CRF with CKD risk were estimated using the RDR derived from the KIHD Study. Summary RRs were pooled using a random effects model to minimize the effect of between-study heterogeneity [60]. Statistical heterogeneity between studies was quantified using standard chi-square tests and the I^2^ statistic [61]. In our pre-specified protocol, we planned to investigate sources of heterogeneity using stratified analysis and random effects meta-regression [62] as well as assess for small study effects using formal tests such as Begg’s funnel plots [63] and Egger’s regression symmetry test [64]. However, these could not be performed because of the limited number of studies (<10). All statistical analyses were conducted using Stata version MP 17 (Stata Corp, College Station, Texas). To grade the quality of the pooled outcome, we used the Grading of Recommendations Assessment, Development and Evaluation (GRADE) tool, a widely adopted reproducible and transparent framework for grading certainty in evidence and used in clinical decision making [65].

## Results

Baseline characteristics and within-person variability in CRF levels

Table1 summarizes the baseline characteristics of the 2,099 participants overall and by CKD development at end of follow-up. In a random subset of 490 participants, CRF levels were re-assessed at 11 years following the baseline measurements. The mean (SD) of baseline and repeat measurements of VO_2peak_ was 30.3 (7.9) and 27.5 (7.0) mL/kg/min, respectively. The mean (SD) of baseline and repeat measurements of CRF expressed in METs was 8.65 (2.27) and 7.85 (2.01), respectively. Overall, the age-adjusted RDR of CRF was 0.59 (95% CI: 0.53 to 0.65), which suggests that the association of CRF with CKD risk using baseline measurements of CRF could under-estimate the true association by [(1/0.59)-1]*100 = 69.5%.

**Table 1:** Baseline participant characteristics overall and according to CKD development

	Overall (*N*=2099)Mean (SD) or median (IQR)	No CKD (*N*=1902)Mean (SD) or median (IQR)	Developed CKD (*N*=197)Mean (SD) or median (IQR)
Cardiorespiratory fitness (METs)	8.65 (2.27)	8.68 (2.26)	8.33 (2.34)
Peak oxygen uptake (mL/kg/min)	30.3 (7.9)	30.4 (7.9)	29.2 (8.2)
*Questionnaire/Prevalent conditions*			
Age at survey (years)	53 (5)	53 (5)	54 (4)
Alcohol consumption (g/week)	32.0 (6.4-94.0)	32.0 (6.4-94.0)	30.6 (5.4-96.0)
Socioeconomic status	8.54 (4.24)	8.45 (4.24)	9.38 (4.20)
History of type 2 diabetes (%)	74 (3.5)	65 (3.4)	9 (4.6)
Current smoking (%)	660 (31.4)	608 (32.0)	52 (26.4)
History of hypertension (%)	624 (29.7)	555 (29.2)	69 (35.0)
History of CHD (%)	500 (23.8)	440 (23.1)	60 (30.5)
*Physical measurements*			
BMI (kg/m^2^)	26.9 (3.5)	26.8 (3.4)	27.7 (3.8)
SBP (mmHg)	134 (17)	134 (17)	137 (18)
DBP (mmHg)	89 (10)	89 (11)	90 (10)
Physical activity (KJ/day)	1183 (621-1938)	1178 (627-1941)	1236 (565-1847)
*Blood-based markers*			
Total cholesterol (mmol/l)	5.92 (1.08)	5.91 (1.08)	6.02 (1.14)
HDL-C (mmol/l)	1.30 (0.30)	1.30 (0.30)	1.29 (0.32)
Serum creatinine (µmol/1)	88.3 (11.6)	88.3 (11.6)	88.1 (12.0)
Estimated GFR (mL/min/1.73 m^2^)	88.0 (16.5)	88.0 (16.6)	87.6 (15.9)

BMI, body mass index; CHD, coronary heart disease; CI, confidence interval; CKD, chronic kidney disease; CRF, cardiorespiratory fitness; DBP, diastolic blood pressure; GFR, glomerular filtration rate; HDL-C, high-density lipoprotein cholesterol; IQR, interquartile range; METs, metabolic equivalents; SD, standard deviation; SBP, systolic blood pressure

### Cardiorespiratory fitness and risk of CKD

#### Prospective cohort analysis

**Fig. 1:**
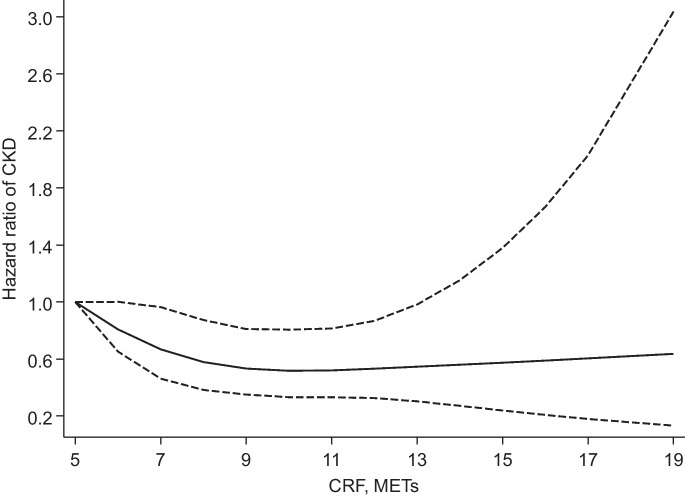
Restricted cubic splines of the hazard ratios of chronic kidney disease with baseline cardiorespiratory fitness. CKD, chronic kidney disease; CRF, cardiorespiratory fitness; Dashed lines represent the 95% confidence intervals for the spline model (solid line). Models were adjusted for age, systolic blood pressure, history of type 2 diabetes, smoking status, history of hypertension, history of coronary heart disease, total cholesterol, alcohol consumption, estimated glomerular filtration rate, physical activity, and socioeconomic status

**Fig. 2:**
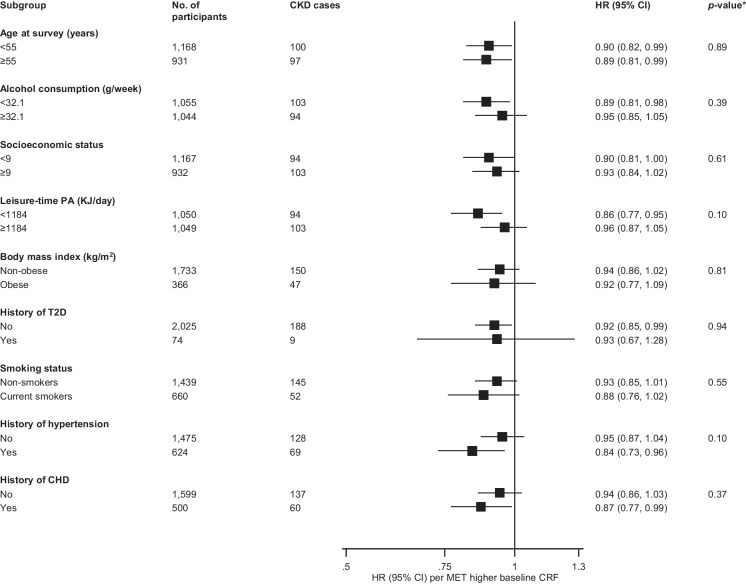
Hazard ratios for baseline values of cardiorespiratory fitness and chronic kidney disease risk by several participant level characteristics. Hazard ratios are adjusted for age, systolic blood pressure, history of type 2 diabetes, smoking status, history of hypertension, history of coronary heart disease, total cholesterol, alcohol consumption, estimated glomerular filtration rate, physical activity, and socioeconomic status; CHD, coronary heart disease; CI, confidence interval; CKD, chronic kidney disease; CRF, cardiorespiratory fitness; HR, hazard ratio; PA, physical activity; SBP, systolic blood pressure; T2D, type 2 diabetes; *, *p*-value for interaction; cut-offs used for age, alcohol consumption, socioeconomic status and physical activity are median values

During a median (interquartile range) follow-up of 25.8 (18.0-28.0) years, 197 incident CKD events (annual rate 4.21/1,000 person-years at risk; 95% CI: 3.66-4.84) were recorded. A multivariable restricted cubic spline curve showed that CKD risk decreased continuously with increasing CRF across the range 5-13 METs (*p*-value for nonlinearity=.17), beyond which the risk remained constant (Fig. 1). The age-adjusted HR (95% CI) for CKD per 1 MET increase in CRF was 0.87 (0.81-0.94) which was minimally attenuated to 0.92 (0.85-0.99) on further adjustment for SBP, history of T2D, smoking status, history of hypertension, history of CHD, total cholesterol, alcohol consumption, estimated GFR, physical activity, and SES. Alternatively, comparing the top versus bottom tertiles of CRF levels, the corresponding adjusted HRs (95% CIs) were 0.53 (0.37-0.76) and 0.67 (0.46-0.97), respectively (Table 2). On correction for regression dilution bias, the HRs were stronger (Table 2). The association did not significantly vary across several clinical subgroups (*p-value* for interaction ≥ .10 for each; Fig. 2).

#### Meta-analysis of published studies

**Fig. 3:**
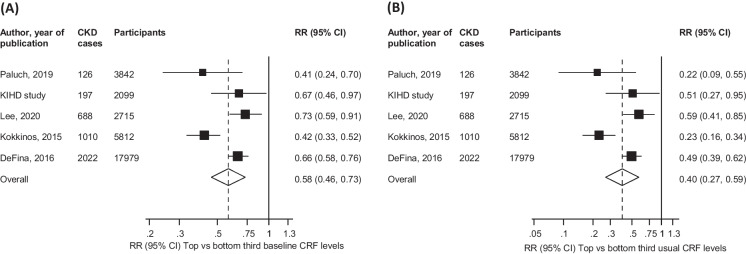
Prospective studies of cardiorespiratory fitness and risk of chronic kidney disease. **A** Using baseline levels of cardiorespiratory fitness; **B** Corrected for regression dilution bias The summary estimates presented were calculated using random effects models; size of a data marker is proportional to the inverse of the variance of the relative ratio; CI, confidence interval (bars); CKD, chronic kidney disease; CRF, cardiorespiratory fitness; RR, relative risk

We identified four general population-based prospective cohort studies reporting on the associations between CRF levels and incident CKD risk (Electronic Supplementary Materials 7**-**8) [20–23]. All four studies were conducted in the United States and assessed CRF following an exercise stress test on a treadmill. Including the current study, the pooled analysis comprised five studies involving 32,447 participants and 4,043 CKD cases. Three of the studies were based in predominantly White individuals and the other two included a mixture of Black and White individuals (approximately 74% Blacks and 26% Whites). The average age at baseline ranged from approximately 25 to 58 years and follow-up duration ranged from 7.2 to 27.9 years. Except for two studies which enrolled only male participants, the rest enrolled both males and females. Using the ROBINS-I tool, all five studies were at serious risk of bias (i.e., were judged to be at serious risk of bias in at least one domain, but not at critical risk of bias in any domain) (Electronic Supplementary Material 9). The pooled RRs (95% CI) for CKD comparing the top versus bottom thirds of baseline and usual levels of CRF in METs in fully-adjusted analyses were 0.58 (0.46-0.73) and 0.40 (0.27-0.59), respectively; (*I*^*2*^=76%, 95% CI: 41 to 90%; *p-value*=.002) (Fig. 3). Exclusion of any single study at a time from the pooled analysis had minimal effect on the pooled RR (Electronic Supplementary Material 10). The GRADE rating for the pooled outcome of CKD was low (Electronic Supplementary Material 11).

## Discussion

### Key findings

Our analysis of this population-based prospective study of middle-aged and older Finnish men showed that high CRF levels were associated with a reduced risk of CKD. The association was independent of several established and emerging risk factors. The risk of CKD decreased in a continuous dose-response fashion with increasing CRF levels from 5-13 METs, beyond which the risk remained constant. There was no evidence of effect modification on the association by several relevant clinical risk markers. On re-evaluation of the relationship between CRF and CKD risk in a larger sample of participants using a pooled analysis of five studies including the new prospective study, there was strong evidence of an association between high CRF levels and reduced CKD risk. On accounting for regression dilution bias using an attenuation factor (RDR) derived from the KIHD study, our results showed that using single baseline measurements of CRF to investigate the association between CRF and CKD risk could under-estimate the true association by about 70%.

### Comparison with previous studies

Though a number of prospective cohort studies have reported on the associations between CRF levels and CKD risk [20–23], certain aspects of the association were not addressed. These included failure to (i) investigate if clinically relevant risk markers could modify the association, (ii) evaluate the detailed dose-response nature of the relationship and (iii) account for changes in levels of CRF due to factors such as random measurement error, ageing, chronic disease and within-person variability. Our findings of a decreased risk of CKD with high CRF levels are consistent with previous studies conducted on the topic [20–23]. In addition to showing that the risk of CKD decreases continuously across the range of CRF values (5-13 METs), we have shown that the association is not modified by important risk factors such as age, BMI, and comorbidities. More importantly, our results show that using only single baseline measurements of CRF could substantially underestimate the CRF-CKD association. Finally, by pooling the findings of all previous studies conducted on the topic which provided enhanced power, our results provide a more precise estimate of the magnitude of the association between CRF levels and CKD risk.

### Possible explanations for findings

About half of the variation in CRF is heritable [66] and it is also influenced by non-modifiable factors such as age, sex and underlying disease states [15]. Nevertheless, CRF remains a modifiable risk factor [67] and the most established ways of increasing CRF levels in healthy individuals or those with prevalent comorbidities are through increased physical activity and exercise training [15]. The protective effects of CRF on CKD risk are likely via the effects of physical activity and exercise training. Physical activity improves metabolic factors such as triglycerides and high-density lipoprotein cholesterol levels, blood pressure, and insulin resistance, as well as reduces the risk of diabetes and hypertension [68, 69], which consequently reduce the risk of CKD. Physical activity may also protect against CKD via (i) improved endothelial function and reduced atherosclerosis of the kidney vasculature which lead to protection against filtration barrier defects, albuminuria, and declining kidney function; (ii) reduction of inflammation and reactive oxygen species; (iii) improved insulin sensitivity; (iv) maintenance of a healthy amount of adipose tissue and reduction in adipocytokines; and (v) alleviation of sympathetic overactivity [70–73].

### Implications of the current findings

These findings add to the existing evidence on the beneficial effects of high levels of CRF on most organ systems. We have also shown that the risk of CKD reduces continuously with increasing CRF until 13 METs; this finding is consistent with a study which showed that mortality reductions with improving CRF persisted until 13 METs [74]. Moderate to high CRF (>8 METs) is known to reduce the risk of adverse cardiovascular outcomes [75]. Physical activity guidelines recommend 150–300 min of moderate intensity physical activity per week, 75–150 min of vigorous intensity physical activity per week, or an equivalent combination of moderate intensity and vigorous intensity physical activity per week for adults (18-64 years), as these are associated with the most health benefits [76]. Though the amount of physical activity needed to achieve certain levels of CRF is not well defined, most middle-aged individuals who meet current recommendations for moderate intensity physical activity are more likely to achieve at least moderate levels of CRF [77, 78]. There are no published physical activity guidelines for the prevention of CKD, however, they do exist for those at high risk of CKD including patients with diabetes and/or hypertension. New guidance issued by the American College of Sports Medicine (ACSM) with regards to exercise/physical activity and T2D recommends engagement in regular aerobic exercise training, the need to reduce sedentary time and to break up sitting time with frequent activity breaks [79]. The new ACSM guidance for hypertension recommends engagement in moderate intensity, aerobic exercise 5-7 d/wk, supplemented by resistance exercise 2-3 d/wk and flexibility exercise ≥2-3 d/wk [80]. Our findings show that levels of CRF substantially decrease in later life (RDR of 0.59) which is due to factors such as ageing, disease and decreased participation in physical activity. Hence, it is crucially important to take into consideration the long-term change in CRF, when assessing its relationship with adverse outcomes such as CKD. Achieving and maintaining the highest level of CRF during adulthood is important for lowering the risk of chronic disease outcomes [81, 82] including CKD. The most established strategy for doing this is through regular and increased physical activity or exercise training. Most countries have established physical activity guidelines and targets. The World Health Organization for example has recommended that all countries implement policies that will enable all people irrespective of age and disability to be physically active [76]. Despite these recommendations, recent global estimates suggest that one in four adults do not meet aerobic exercise recommendations and global levels of participation in physical activity have shown no overall improvement over the last twenty years [83]. There is also evidence of inequalities in participation in physical activity by age, gender, disability, SES, and geographical location [84, 85]. Chronic kidney disease is a growing global epidemic and a major public health burden. There is an urgent need to invest in services that promote physical activity across all sectors and also to address the inequalities in participation. Major guideline bodies should also consider developing specific physical activity recommendations for CKD prevention.

There is no doubt that physical activity confers protection for a multitude of health outcomes and is a potentially important strategy for reducing the incidence of CKD. Indeed, recent results from an ancillary analysis of a randomized clinical trial showed that when compared with health education, a physical activity and exercise intervention slowed the rate of decline in estimated GFR among community-dwelling sedentary older adults [86]. However, a multifaceted approach is needed for the primary prevention of CKD. Other strategies include maintaining a healthy weight, good nutrition, avoiding the use of tobacco, screening of high-risk individuals such as those with hypertension and diabetes, and raising CKD awareness among the public and policy makers [87]. It has been reported that nutrition therapy has a potential role in increasing levels of CRF in multiple population groups with exercise limitations [15]; this is an area for further investigation.

### Strengths and limitations

In a single study, we have assessed the nature and magnitude of the association between CRF and incident CKD using a large-scale population-based prospective cohort study and pooled analysis of previously published cohort studies including the current study. Strengths of the primary cohort study were the inclusion of a general population-based sample of middle-aged and older men who were nationally representative; the long-follow period and zero loss to follow-up; and comprehensive analyses including adjustment for several established risk factors, evaluation of the dose-response relationship and assessment for effect modification using several clinically relevant characteristics (subgroups). Furthermore, repeat measurements of CRF made within a relatively large random sample of individuals 11 years after baseline were available, which enabled correction for the extent of within-person variability in CRF levels over the long period of follow-up. We were unable to perform a time-varying analysis to allow for changes in CRF given that repeat measurements were only available in a subset of participants. It has been shown that corrections using the RDR could result in overcorrection of the risk estimates if the relationship between the exposure and outcome is not short term [88]. The current risk estimate should therefore be interpreted with caution as there is a possibility that the true estimate lies between that of the time-fixed analysis without and with correction for regression dilution. An important strength of the meta-analysis was the ability to transform risk estimates into a consistent comparison (tertiles), which enabled pooling. Limitations of the primary cohort study included (i) findings being only generalizable to middle-aged and older Caucasian men; (ii) potential for biases such as reverse causation and residual confounding given the observational cohort design; (iii) absence of data on the precise cause of CKD and classification of CKD; and (iv) inability to establish cause and effect. We also acknowledge that estimated GFR (as measured by the CKD-EPI equation) is more accurate for values >60 mL/min/1.73 m^2^; hence, may not be an optimal marker of renal function in individuals with CKD, especially those with concomitant malnutrition and/or sarcopenia. However, the mean estimated GFR in the study population was 88.0 mL/min/1.73 m^2^. The KIHD recruited approximately general population participants, hence, it is unlikely individuals with malnutrition or sarcopenia were included. The pooled analysis was limited by the limited number of studies published on the topic, inability to fully examine the impact of consistent adjustment for potential confounders because of variable adjustment by studies, and the fact that we used the RDR derived from the KIHD in correcting the pooled estimate for regression dilution bias.

## Conclusion

Findings from a new prospective study and pooled analysis of previous studies plus the new study show that high CRF levels are independently associated with a reduced risk of CKD, consistent with a linear dose-response relationship. Using single baseline measurements of CRF to investigate the association between CRF and CKD risk could considerably under-estimate the true association. Strategies that can increase or maintain high levels of CRF such as regular aerobic physical activity and exercise training should be encouraged via population wide approaches and across all sectors.

## Supplementary information

ESM 1
