# Supplementary material for: Baseline and usual cardiorespiratory fitness and the risk of chronic kidney disease: A prospective study and meta-analysis of published observational cohort studies
Source: GeroScience. 2023 Jan 17;45(3):1761–74. doi: 10.1007/s11357-023-00727-3 (PMC10400499; doi:10.1007/s11357-023-00727-3)
Supplement: Supplementary file 1 — ESM 1 [file 11357_2023_727_MOESM1_ESM.docx]

**ELECTRONIC SUPPLEMENTARY MATERIAL**

**Baseline and long-term cardiorespiratory fitness and the risk of chronic kidney disease: A new prospective study and meta-analysis of published observational cohort studies**

| **Electronic Supplementary Material 1** | STROBE Statement |
| --- | --- |
| **Electronic Supplementary Material 2** | Variables with missing data |
| **Electronic Supplementary Material 3** | Participant flow |
| **Electronic Supplementary Material 4** | PRISMA checklist |
| **Electronic Supplementary Material 5** | MOOSE checklist |
| **Electronic Supplementary Material 6** | Literature search strategy |
| **Electronic Supplementary Material 7** | Flow of studies included in pooled analysis |
| **Electronic Supplementary Material 8** | Baseline characteristics of studies included in pooled analysis |
| **Electronic Supplementary Material 9** | Risk of bias assessment |
| **Electronic Supplementary Material 10** | Relative risks on exclusion of a study one at a time |
| **Electronic Supplementary Material 11** | GRADE summary of findings |

**Electronic Supplementary Material 1** STROBE Statement

| **Section/Topic** | Item # | Recommendation | Reported on page # |
| --- | --- | --- | --- |
| **Title and abstract** | 1 | (*a*) Indicate the study’s design with a commonly used term in the title or the abstract | Page 1 |
|  |  | (*b*) Provide in the abstract an informative and balanced summary of what was done and what was found | Page 2 |
| Introduction | | |  |
| Background/rationale | 2 | Explain the scientific background and rationale for the investigation being reported | Introduction |
| Objectives | 3 | State specific objectives, including any pre-specified hypotheses | Introduction |
| Methods | | |  |
| Study design | 4 | Present key elements of study design early in the paper | Study design and participants |
| Setting | 5 | Describe the setting, locations, and relevant dates, including periods of recruitment, exposure, follow-up, and data collection | Study design and participants |
| Participants | 6 | (*a*) Give the eligibility criteria, and the sources and methods of selection of participants. Describe methods of follow-up | Study design and participants |
|  |  | (*b*) For matched studies, give matching criteria and number of exposed and unexposed | Not applicable |
| Variables | 7 | Clearly define all outcomes, exposures, predictors, potential confounders, and effect modifiers. Give diagnostic criteria, if applicable | Assessment of CRF and other covariates |
| Data sources/ measurement | 8* | For each variable of interest, give sources of data and details of methods of assessment (measurement). Describe comparability of assessment methods if there is more than one group | Assessment of CRF and other covariates |
| Bias | 9 | Describe any efforts to address potential sources of bias | Statistical analyses |
| Study size | 10 | Explain how the study size was arrived at | Statistical analyses |
| Quantitative variables | 11 | Explain how quantitative variables were handled in the analyses. If applicable, describe which groupings were chosen and why | Statistical analyses |
| Statistical methods | 12 | (*a*) Describe all statistical methods, including those used to control for confounding | Statistical analyses |
|  |  | (*b*) Describe any methods used to examine subgroups and interactions | Statistical analyses |
|  |  | (*c*) Explain how missing data were addressed | Not applicable |
|  |  | (*d*) If applicable, explain how loss to follow-up was addressed | Not applicable |
|  |  | (*e*) Describe any sensitivity analyses | Statistical analyses |
| Results | | |  |
| Participants | 13* | (a) Report numbers of individuals at each stage of study—eg numbers potentially eligible, examined for eligibility, confirmed eligible, included in the study, completing follow-up, and analysed | Study design and participants |
|  |  | (b) Give reasons for non-participation at each stage | Study design and participants |
|  |  | (c) Consider use of a flow diagram | ESM 3 |
| Descriptive data | 14* | (a) Give characteristics of study participants (eg demographic, clinical, social) and information on exposures and potential confounders | Results; Tables 1 |
|  |  | (b) Indicate number of participants with missing data for each variable of interest |  |
|  |  | (c) Summarise follow-up time (eg, average and total amount) | Results |
| Outcome data | 15* | Report numbers of outcome events or summary measures over time | Results |
| Main results | 16 | (*a*) Give unadjusted estimates and, if applicable, confounder-adjusted estimates and their precision (eg, 95% confidence interval). Make clear which confounders were adjusted for and why they were included | Results; Table 2 |
|  |  | (*b*) Report category boundaries when continuous variables were categorized | Results; Table 2 |
|  |  | (*c*) If relevant, consider translating estimates of relative risk into absolute risk for a meaningful time period |  |
| Other analyses | 17 | Report other analyses done—eg analyses of subgroups and interactions, and sensitivity analyses | Results; Figure 2 |
| Discussion |  |  |  |
| Key results | 18 | Summarise key results with reference to study objectives | Discussion - Summary of main findings |
| **Limitations** |  |  |  |
| Interpretation | 20 | Give a cautious overall interpretation of results considering objectives, limitations, multiplicity of analyses, results from similar studies, and other relevant evidence | Discussion |
| Generalisability | 21 | Discuss the generalisability (external validity) of the study results | Discussion |
| Other information |  |  |  |
| Funding | 22 | Give the source of funding and the role of the funders for the present study and, if applicable, for the original study on which the present article is based | After Acknowledgement |

**Electronic Supplementary Material 2** Variables with missing data

| **Variable** | **Missing** | **Total** | **Percent missing** |
| --- | --- | --- | --- |
| Cardiorespiratory fitness (METs) | 304 | 2626 | 11.58 |
| Age | 0 | 2626 | 0.00 |
| Systolic blood pressure | 14 | 2626 | 0.53 |
| History of hypertension | 0 | 2626 | 0.00 |
| History of CHD | 0 | 2626 | 0.00 |
| Smoking status | 4 | 2626 | 0.15 |
| History of type 2 diabetes | 0 | 2626 | 0.00 |
| Total cholesterol | 50 | 2626 | 1.90 |
| Alcohol consumption | 4 | 2626 | 0.15 |
| Estimated GFR | 216 | 2626 | 8.23 |
| Socioeconomic status | 0 | 2626 | 0.00 |
| Physical activity | 26 | 2626 | 0.99 |

CHD, coronary heart disease; GFR, glomerular filtration rate

**Electronic Supplementary Material 3** Participant flow

CKD, chronic kidney disease; CRF, cardiorespiratory fitness

**Electronic Supplementary Material 4** PRISMA checklist

| **Section/topic** | **Item No** | **Checklist item** | **Reported on page No** |
| --- | --- | --- | --- |
| **Title** | | | |
| Title | 1 | Identify the report as a systematic review, meta-analysis, or both | 1 |
| **Abstract** | | | |
| Structured summary | 2 | Provide a structured summary including, as applicable, background, objectives, data sources, study eligibility criteria, participants, interventions, study appraisal and synthesis methods, results, limitations, conclusions and implications of key findings, systematic review registration number | 2 |
| **Introduction** | | | |
| Rationale | 3 | Describe the rationale for the review in the context of what is already known | Introduction |
| Objectives | 4 | Provide an explicit statement of questions being addressed with reference to participants, interventions, comparisons, outcomes, and study design (PICOS) | Introduction |
| **Methods** | | | |
| Protocol and registration | 5 | Indicate if a review protocol exists, if and where it can be accessed (such as web address), and, if available, provide registration information including registration number | Methods |
| Eligibility criteria | 6 | Specify study characteristics (such as PICOS, length of follow-up) and report characteristics (such as years considered, language, publication status) used as criteria for eligibility, giving rationale | Methods |
| Information sources | 7 | Describe all information sources (such as databases with dates of coverage, contact with study authors to identify additional studies) in the search and date last searched | Methods |
| Search | 8 | Present full electronic search strategy for at least one database, including any limits used, such that it could be repeated | ESM 6 |
| Study selection | 9 | State the process for selecting studies (that is, screening, eligibility, included in systematic review, and, if applicable, included in the meta-analysis) | Methods |
| Data collection process | 10 | Describe method of data extraction from reports (such as piloted forms, independently, in duplicate) and any processes for obtaining and confirming data from investigators | Methods |
| Data items | 11 | List and define all variables for which data were sought (such as PICOS, funding sources) and any assumptions and simplifications made | Methods |
| Risk of bias in individual studies | 12 | Describe methods used for assessing risk of bias of individual studies (including specification of whether this was done at the study or outcome level), and how this information is to be used in any data synthesis | Methods |
| Summary measures | 13 | State the principal summary measures (such as risk ratio, difference in means). | Methods |
| Synthesis of results | 14 | Describe the methods of handling data and combining results of studies, if done, including measures of consistency (such as I^2^ statistic) for each meta-analysis | Methods |
| Risk of bias across studies | 15 | Specify any assessment of risk of bias that may affect the cumulative evidence (such as publication bias, selective reporting within studies) | Methods |
| Additional analyses | 16 | Describe methods of additional analyses (such as sensitivity or subgroup analyses, meta-regression), if done, indicating which were pre-specified | Methods |
| **Results** | | | |
| Study selection | 17 | Give numbers of studies screened, assessed for eligibility, and included in the review, with reasons for exclusions at each stage, ideally with a flow diagram | ESM 7 |
| Study characteristics | 18 | For each study, present characteristics for which data were extracted (such as study size, PICOS, follow-up period) and provide the citations | ESM 8 |
| Risk of bias within studies | 19 | Present data on risk of bias of each study and, if available, any outcome-level assessment (see item 12). | ESM 9 |
| Results of individual studies | 20 | For all outcomes considered (benefits or harms), present for each study (a) simple summary data for each intervention group and (b) effect estimates and confidence intervals, ideally with a forest plot | Figure 3 |
| Synthesis of results | 21 | Present results of each meta-analysis done, including confidence intervals and measures of consistency | Results and Figure 3 |
| Risk of bias across studies | 22 | Present results of any assessment of risk of bias across studies (see item 15) | ESM 8 |
| Additional analysis | 23 | Give results of additional analyses, if done (such as sensitivity or subgroup analyses, meta-regression) (see item 16) | ESM 10 |
| **Discussion** | | | |
| Summary of evidence | 24 | Summarise the main findings including the strength of evidence for each main outcome; consider their relevance to key groups (such as health care providers, users, and policy makers) | Discussion |
| Limitations | 25 | Discuss limitations at study and outcome level (such as risk of bias), and at review level (such as incomplete retrieval of identified research, reporting bias) | Discussion |
| Conclusions | 26 | Provide a general interpretation of the results in the context of other evidence, and implications for future research | Discussion |
| **Funding** | | | |
| Funding | 27 | Describe sources of funding for the systematic review and other support (such as supply of data) and role of funders for the systematic review | None |

**Electronic Supplementary Material 5** MOOSE checklist

| **Criteria** | | **Brief description of how the criteria were handled in the review** |
| --- | --- | --- |
| **Reporting of background** | |  |
| √ | Problem definition | Previous studies may have underestimated the association between cardiorespiratory fitness (CRF) and risk of chronic kidney disease (CKD) |
| √ | Hypothesis statement | There magnitude of the association between CRF and CKD may be stronger than previously reported. |
| √ | Description of study outcomes | CKD |
| √ | Type of exposure | CRF |
| √ | Type of study designs used | Prospective (cohort, case-cohort or “nested case control”) population-based studies |
| √ | Study population | Approximately general populations |
| **Reporting of search strategy should include** | |  |
| √ | Qualifications of searchers | Setor Kunutsor, MD PhD; Nzechukwu M. Isiozor, MD PhD |
| √ | Search strategy, including time period included in the synthesis and keywords | Time period: from inception of MEDLINE and Embase to 22 May 2022  **Search strategy:**  In ESM 6 |
| √ | Databases and registries searched | MEDLINE and Embase |
| √ | Search software used, name and version, including special features | Ovid was used to search Embase  Endnote used to manage references |
| √ | Use of hand searching | We searched bibliographies of retrieved papers and Web of Science citation search |
| √ | List of citations located and those excluded, including justifications | Details of the literature search process are outlined in the flow chart. The citation list for excluded studies is available upon request. |
| √ | Method of addressing articles published in languages other than English | We placed no restrictions on language |
| √ | Method of handling abstracts and unpublished studies | Not applicable |
| √ | Description of any contact with authors | Not applicable |
| **Reporting of methods should include** | |  |
| √ | Description of relevance or appropriateness of studies assembled for assessing the hypothesis to be tested | Detailed inclusion and exclusion criteria are described in the Methods section. |
| √ | Rationale for the selection and coding of data | Data extracted from each of the studies were relevant to the population characteristics, study design, exposure, outcome, and possible effect modifiers of the association. |
| √ | Assessment of confounding | We assessed confounding by ranking individual studies on the basis of different adjustment levels. |
| √ | Assessment of study quality, including blinding of quality assessors; stratification or regression on possible predictors of study results | The Cochrane Risk of Bias in Non-randomised Studies – of Interventions (ROBINS-I) tool was used to assess the risk of bias within individual observational studies |
| √ | Assessment of heterogeneity | Heterogeneity of the studies was explored with I^2^ statistic that provides the relative amount of variance of the summary effect due to the between-study heterogeneity. |
| √ | Description of statistical methods in sufficient detail to be replicated | Description of methods of meta-analyses, sensitivity analyses, meta-regression and assessment of publication bias are detailed in the methods. We performed random effects meta-analysis with Stata MP 17 |
| √ | Provision of appropriate tables and graphics | Figure 3; ESM 7, 8, 9, 10, 11 |
| **Reporting of results should include** | |  |
| √ | Graph summarizing individual study estimates and overall estimate | Figure 3 |
| √ | Table giving descriptive information for each study included | ESM 8 |
| √ | Results of sensitivity testing | Sensitivity analysis was conducted to assess the influence of each individual study by omitting one study at a time and calculating a pooled estimate for the remainder of the studies (ESM 10) |
| √ | Indication of statistical uncertainty of findings | 95% confidence intervals were presented with all summary estimates and I^2^ values |
| **Reporting of discussion should include** | |  |
| √ | Quantitative assessment of bias | Publication bias could not be assessed because of the few studies. |
| √ | Justification for exclusion | All studies were excluded based on the pre-defined inclusion criteria in methods section. |
| √ | Assessment of quality of included studies | Brief discussion included in ‘Methods’ section |
| **Reporting of conclusions should include** | |  |
| √ | Consideration of alternative explanations for observed results | Discussed in the context of the results. |
| √ | Generalization of the conclusions | Discussed in the context of the results. |
| √ | Guidelines for future research | Further large-scale studies are needed |
| √ | Disclosure of funding source | None |

**Electronic Supplementary Material 6** Literature search strategy

| Relevant studies, published before 22 May 2022 (date last searched), were identified through electronic searches using MEDLINE and Embase databases. Electronic searches were supplemented by scanning reference lists of articles identified for all relevant studies (including review articles) and the “Cited Reference Search” function in Web of Science. The computer-based searches combined search terms related to cardiorespiratory fitness and chronic kidney disease without language restriction.  1 exp Physical Fitness/ or exp Cardiorespiratory Fitness/ (34854)  2 aerobic fitness.mp. (3539)  3 cardiovascular fitness.mp. (1464)  4 aerobic capacity.mp. (5909)  5 exercise capacity.mp. (14621)  6 fitness.mp. (104121)  7 cardio fitness.mp. (12)  8 oxygen uptake.mp. (21942)  9 chronic kidney disease.mp. or exp Renal Insufficiency, Chronic/ (159066)  10 kidney failure.mp. (106402)  11 exp Kidney Diseases/ (554420)  12 kidney insufficiency.mp. (742)  13 renal disease.mp. (65037)  14 renal failure.mp. (93518)  15 exp Proteinuria/ (41340)  16 cohort studies/ or longitudinal studies/ or follow-up studies/ or prospective studies/ or retrospective studies/ or cohort.ti,ab. or longitudinal.ti,ab. or prospective.ti,ab. or retrospective.ti,ab. (3033067)  17 1 or 2 or 3 or 4 or 5 or 6 or 7 or 8 (141026)  18 9 or 10 or 11 or 12 or 13 or 14 or 15 (640388)  19 16 and 17 and 18 (235)  20 limit 19 to (humans and ("young adult (19 to 24 years)" or "adult (19 to 44 years)" or "young adult and adult (19-24 and 19-44)" or "middle age (45 to 64 years)" or "middle aged (45 plus years)" or "all aged (65 and over)" or "aged (80 and over)")) (194)  ***********************  Each part was specifically translated for searching the other databases |
| --- |

**Electronic Supplementary Material 7** Flow of studies included in meta-analysis

**Electronic Supplementary Material 8** Baseline characteristics of studies included in pooled analysis

| **Author, year of publication** | **Study name** | **Country** | **Race (%)** | **Baseline year** | **Baseline age, years** | **Males, %** | **Follow-up, years** | **CRF assessment** | **CKD cases** | **No. of participants** | **Adjustment factors** |
| --- | --- | --- | --- | --- | --- | --- | --- | --- | --- | --- | --- |
| Kokkinos, 2015 | Veterans Affairs Medical Center | USA | Black (73.8%)  White (26.2%) | 1987-2012 | 58.4 | 100.0 | 8.0 | Maximal | 1010 | 5812 | Age, baseline estimated GFR, BMI, race, b-blockers, calcium-channel blockers, ACE-I/ARBs, diuretics, hypoglycaemic and lipid-lowering agents, history of smoking, and history of alcohol abuse, sleep apnoea, CVD, dyslipidaemia, hypertension, and DM |
| DeFina, 2016 | CCLS | USA | Non-Hispanic White | 1971-2009 | 50.0 | 78.0 | 7.2 | Maximal | 2022 | 17979 | Age at baseline, sex, year of baseline visit, self-reported hypertension, SBP, fasting glucose, self-reported hyperlipidaemia, and TC |
| Paluch, 2019 | CARDIA | USA | Black (50.5%)  White (49.5%) | 1985-1986 | 24.8 | 46.5 | 27.9 | Maximal | 126 | 3842 | Gender, race, age, maximal education throughout study, field center, time varying healthy eating index score, smoking status, alcohol intake, time varying BMI, SBP, fasting glucose, and baseline estimated GFR |
| Lee, 2020 | Framingham Offspring Study | USA | White | 1978-1982 | 43.0 | 48.5 | 24.8 | Submaximal | 688 | 2715 | Age, sex, estimated GFR, resting HR, current smoking status, BMI, TC to HDL-C ratio, SBP, DBP, blood glucose level, DM, and use of antihypertensive and lipid-lowering medications |
| Current study | KIHD | Finland | White | 1984-1989 | 53.0 | 100.0 | 25.8 | Maximal | 197 | 2099 | Age, SBP, history of T2D, smoking status, history of hypertension, history of CHD, TC, alcohol consumption, estimated GFR, physical activity, and SES |

ACE-I, angiotensin-converting enzyme inhibitor; ARB, angiotensin receptor blocker; BMI, body mass index; CHD, coronary heart disease; CKD, chronic kidney disease; CRF, cardiorespiratory fitness; CVD, cardiovascular disease; DBP, diastolic blood pressure; DM, diabetes mellitus; GFR, glomerular filtration rate; HDL-C, high-density lipoprotein cholesterol; HR, heart rate; SBP, systolic blood pressure; SES, socioeconomic status; T2D, type 2 diabetes; TC, total cholesterol

Study Abbreviations: CARDIA, Coronary Artery Risk Development in Young Adults; CCLS, Cooper Center Longitudinal Study; KIHD, Kuopio Ischemic Heart Disease

**Electronic Supplementary Material 9** Risk of bias assessment

**Electronic Supplementary Material 10** Relative risks on exclusion of a study one at a time

CI, confidence interval

**Electronic Supplementary Material 11** GRADE summary of findings

| **Outcomes** | **№ of participants (studies) Follow-up** | **Certainty of the evidence (GRADE)** | **Relative effect (95% CI)** | **Anticipated absolute effects** | |
| --- | --- | --- | --- | --- | --- |
|  |  |  |  | **Risk with Low CRF** | **Risk difference with High CRF** |
| Risk of CKD in overall population | (5 observational studies) | ⨁⨁◯◯ Low^a,b^ | **RR 0.58** (0.46 to 0.73) | 0 per 1,000 | **0 fewer per 1,000** (0 fewer to 0 fewer) |
| *The risk in the intervention group (and its 95% confidence interval) is based on the assumed risk in the comparison group and the relative effect of the intervention (and its 95% CI).  CI, confidence interval; CKD, chronic kidney disease; CRF, cardiorespiratory fitness; RR, risk ratio | | | | | |
| **GRADE Working Group grades of evidence** **High certainty:** we are very confident that the true effect lies close to that of the estimate of the effect. **Moderate certainty:** we are moderately confident in the effect estimate: the true effect is likely to be close to the estimate of the effect, but there is a possibility that it is substantially different. **Low certainty:** our confidence in the effect estimate is limited: the true effect may be substantially different from the estimate of the effect. **Very low certainty:** we have very little confidence in the effect estimate: the true effect is likely to be substantially different from the estimate of effect. | | | | | |

**Explanations**

a. One or more studies were at overall serious risk of bias

b. I-squared value of 76%
